# High prevalence of Hb Q-Thailand not in cis with the -α^4.2^ deletion: genotypes, phenotypes, and implications in the cenxi population of southern China

**DOI:** 10.3389/fgene.2026.1824951

**Published:** 2026-05-11

**Authors:** Yanwei Li, Meiling Wu, Lihong Zheng, Liang Liang, Youqiong Li

**Affiliations:** 1 Department of Clinical Laboratory, CenXi Maternity & Child Healthcare Hospital, Wuzhou, China; 2 Center for Medical Genetics and Prenatal Diagnosis, People’s Hospital of Guangxi Zhuang Autonomous Region, Nanning, China

**Keywords:** cenxi, Hb Q-Thailand, phenotypes, thalassemia, -α4.2

## Abstract

**Objectives:**

Hb Q-Thailand, a common hemoglobin variation in Southeast Asia, has historically been associated with the -α^4.2^ deletion. However, in the Cenxi population of southern China, this variant is frequently detected without the -α^4.2^ deletion. This study aimed to investigate the carrier rate, genotype distribution, and phenotypic characteristics of Hb Q-Thailand not associated with the -α^4.2^ deletion in this population.

**Methods:**

A total of 23,546 individuals who underwent capillary electrophoresis screening at our hospital between January 2024 and December 2025 were enrolled in this study. Deletional α-thalassemia mutations were detected using Gap-PCR, while common α-globin chain mutations were identified by PCR-reverse dot blot hybridization (PCR-RDB). Additionally, 17 common β-thalassemia point mutations were analyzed using PCR-RDB. Sanger sequencing was performed to characterize α-globin variants.

**Results:**

Thirty-seven positive cases were identified among the 23,546 screened individuals, yielding a positive screening rate of 0.16% (37/23,546) in the Cenxi population. Genetic confirmation was performed in 22 cases, of which 21 were confirmed to carry Hb Q-Thailand, while the remaining case was identified as a rare variant, Hb Zhaoqing. Notably, 10 samples exhibited Hb Q-Thailand without linkage to the -α^4.2^ deletion, accounting for a substantial proportion of 47.6% (10/21) among confirmed cases. In the group with Hb Q-Thailand not linked to the -α^4.2^ deletion, hematological phenotypes were largely within normal ranges. The Hb Q-Thailand level in simple heterozygotes was 16.6% ± 0.4%, which increased to 22.4% ± 0.4% when co-inherited with the -α^3.7^ deletion. In contrast, the majority of individuals in the group with Hb Q-Thailand linked to the -α^4.2^ deletion exhibited abnormal hematological parameters. The Hb Q-Thailand level in this group was 28.2% ± 0.8%, rising to 42.0% when the -α^3.7^ deletion was co-inherited. Additionally, one case in this group was found to co-inherit β-thalassemia trait, presenting with hematological and electrophoretic features consistent with β-thalassemia trait, characterized by elevated Hb A_2_ and reduced MCV/MCH.

**Conclusion:**

The Cenxi population presents a high proportion of Hb Q-Thailand cases in which the variant is not linked to the -α^4.2^ deletion. Hematological phenotypes differ significantly depending on the presence or absence of linkage to the -α^4.2^ deletion.

## Introduction

Hemoglobinopathies represent the most common group of monogenic disorders and are broadly classified into two categories: thalassemias and structural hemoglobin (Hb) variants (Fucharoen and Winichagoon, 2002). Thalassemias are characterized by reduced or absent synthesis of normal globin chains, whereas structural Hb variants result from amino acid substitutions within the globin chains. These disorders are widely distributed across Africa, Mediterranean countries, and Asia (Goonasekera et al., 2018; Paiboonsukwong et al., 2022; Musallam et al., 2023). In southern China, the most prevalent deletional α-thalassemia mutations include--^SEA^, -α^4.2^, and -α^3.7^, while the most common structural α-globin chain variants are Hb Constant Spring (Hb CS), Hb Westmead (Hb WS), and Hb Quong Sze (Hb QS) (Huang et al., 2019). Collectively, these mutations account for approximately 95% of α-globin abnormalities in this region.

Hb Q-Thailand is an α-globin chain variant arising from a missense mutation at codon 74 of the α1-globin gene (*HBA1*) on chromosome 16, resulting in the substitution of aspartic acid by histidine at position 74 of the α-globin peptide (Zeng et al., 1992). As this alteration occurs in the external region (EF3) of the hemoglobin molecule, it typically does not affect structural stability or oxygen-carrying capacity. Consequently, carriers—particularly heterozygotes—are usually asymptomatic and exhibit normal hematological parameters. This often leads to Hb Q-Thailand being overlooked during hemoglobin screening strategies. Although not detectable by routine blood cell analysis, Hb Q-Thailand can be identified through distinct abnormal peaks observed in high-performance liquid chromatography or capillary electrophoresis.

In regions with a high prevalence of hemoglobinopathies, such as southern China and Southeast Asia, Hb Q-Thailand exhibits a considerable carrier frequency and is almost invariably linked in *cis* to the -α^4.2^ deletion (Singsanan et al., 2010; Li et al., 2016). When co-inherited with α^0^-thalassemia, this variant can give rise to a complex thalassemia phenotype known as Hb Q-H disease (Hu et al., 2011). Rare cases of Hb Q-Thailand not linked to the -α^4.2^ deletion have been documented sporadically (Qin et al., 2023). However, in the Cenxi region of Guangxi, this unlinked inheritance pattern appears to be relatively common. In the present study, we aimed to investigate the carrier rate, phenotypic features, and genotypic characteristics of Hb Q-Thailand not associated with the -α^4.2^ deletion in this population.

## Materials and methods

### Sample

**FIGURE 1:**
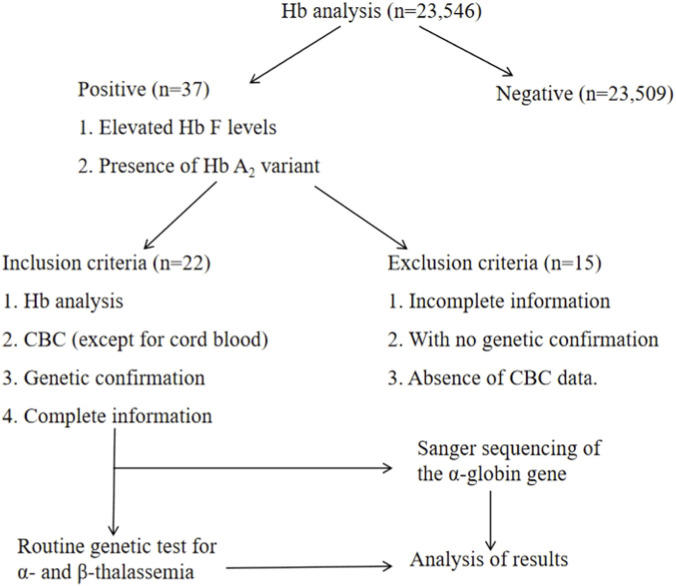
Study flowchart of this study.

A total of 23,546 individuals from the Cenxi region who underwent thalassemia screening at our hospital between January 2024 and December 2025 were enrolled in this study. In adults, a positive screening result by capillary electrophoresis (CE) was defined by the presence of an isolated Hb A_2_ variant and elevated values in the Hb F/Q zone (suspected hemoglobin variants). In neonatal cord blood, positivity was indicated by a slow-migrating band in the Hb A_2_ region showing an atypical migration pattern on CE, suggestive of a hemoglobin variant. The inclusion criteria were: (1) positive CE screening; (2) available complete blood count (CBC) results (except for cord blood); (3) genetic confirmation; and (4) complete individual basic information. Exclusion criteria comprised (1) incomplete information; (2) lack of genetic confirmation; and (3) absence of CBC data. The study flowchart is shown in Figure 1. This study was approved by the Ethics Committee of Cenxi Maternal and Child Health Hospital (2026030102). Written informed consent was obtained from all participants prior to their inclusion.

### Hematological parameters and Hb analysis

Hematological parameters were obtained using an automated blood cell analyzer (XN-10B3; Sysmex, Kobe, Japan). The observed indicators included hemoglobin (Hb), mean corpuscular volume (MCV), and mean corpuscular hemoglobin (MCH), with reference ranges defined as follows: Hb 115–160 g/L for females and 120–165 g/L for males; MCV 82–100 fL; and MCH 27–35 pg. Hemoglobin fraction separation and quantification were performed using CE (V8; Helena, Beaumont, United States). The reference ranges for healthy individuals were as follows: Hb A_2_ 2.4%–3.5%, Hb F 0%–5%, and Hb A 91.5%–97.5%. Reference ranges for Hb analysis in neonatal cord blood: Hb A 4%–40%, Hb F 60%–96%. Based on the Hb A_2_ levels, the direction of genetic analysis was determined: when Hb A_2_ was below 2.4%, α-thalassemia genetic testing was conducted, whereas when it exceeded 3.5%, both α- and β-thalassemia genetic analyses were performed. In cases where δ-thalassemia was present, the measured Hb A_2_ level could fall within the normal reference range, thereby potentially obscuring the diagnosis of concomitant β-thalassemia. Consequently, in regions where thalassemia was highly prevalent, patients were routinely screened for both α- and β-thalassemia concurrently to minimize the risk of missed diagnoses.

### Routine genetic tests for thalassemia

In accordance with the kit procedure, genomic DNA was isolated from peripheral blood (Yaneng Biotechnology Company, Shenzhen, China). The four most common types of deletional α-thalassemia in the Chinese population were identified using the gap-polymerase chain reaction (Gap-PCR): -^SEA^, --^THAI^, -α^3.7^, and -α^4.2^ (Yilifang Biotechnology Company, Shenzhen, China). Three frequent mutations of the α-globin gene were identified using PCR and reverse dot blot (PCR-RDB): Hb Westmead (Hb WS), Hb Quong Sze (Hb QS), and Hb Constant Spring (Hb CS) (Yaneng Biotechnology Company, Shenzhen, China). PCR-RDB (Yaneng Biotechnology Company, Shenzhen, China) was used to analyze the 17 known β-thalassemia mutations, which included −32 (C→A), −30 (T→C), −29 (A→G), −28 (A→G), CD14/15 (+G), CD17 (A→T), CD26 (G→A) (Hb E), CD27/28 (+C), CD31 (-C), CD41/42 (-TTCT), CD43 (G→T), CD71/72 (+A), IVS-I-1 (G→T), IVS-I-5 (G→C), IVS-II-654 (C→T), 5′UTR+40–43 (-AAAC) (CAP), and Initiation codon (ATG→ACG).

### Sanger sequencing of the α-globin gene

Suspected positive samples exhibiting electrophoretic patterns consistent with α-globin chain variants were subjected to Sanger sequencing for confirmation. Based on previously reported protocols (Li et al., 2018), primers were designed to amplify the *HBA* genes, and sequence analysis was performed on a 3500XL automated genetic analyzer (ABI, Foster City, CA, United States). The obtained sequences were then aligned against the reference sequences for mutation identification.

## Results

### Prevalence of Hb Q-Thailand in the Cenxi region

**FIGURE 2:**
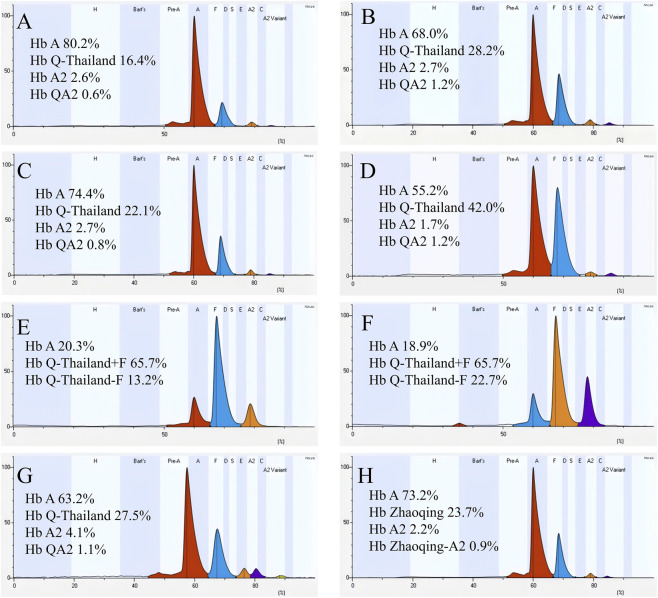
Schematic representation of capillary electrophoresis profiles for different genotypes: **(A)** Hb Q-Thailand not linked to the -α^4.2^ deletion; **(B)** Hb Q-Thailand linked to the -α^4.2^ deletion; **(C)** Hb Q-Thailand not linked to the -α^4.2^ deletion with co-inheritance of the -α^3.7^ deletion; **(D)** Hb Q-Thailand linked to the -α^4.2^ deletion with co-inheritance of the -α^3.7^ deletion; **(E)** Cord blood sample with Hb Q-Thailand not linked to the -α^4.2^ deletion; **(F)** Cord blood sample with Hb Q-Thailand linked to the -α^4.2^ deletion; **(G)** Hb Q-Thailand linked to the -α^4.2^ deletion with co-inheritance of IVS-II-654 C>T mutation.

In this study, a total of 37 samples met the inclusion criteria, yielding a positive screening rate of 0.16% (37/23,546) for CE in the Cenxi population. CE revealed distinct fractionation into 14 zones, with an elevated abnormal peak observed in Zone 9—a region corresponding to the migration positions of Hb F or Hb Q (Figure 2). Among these 37 samples, the vast majority were peripheral blood specimens, with only two identified as cord blood samples. Of the 22 samples that underwent routine thalassemia genetic testing and Sanger sequencing for confirmation, 21 (10 males and 11 females) were found to carry Hb Q-Thailand (Table 1).

**TABLE 1:** The phenotypes and genotypes of 22 patients in this study.

Genotype	Number	Age (years)	Hb (g/dL)	MCV (fL)	MCH (pg)	HbA (%)	Hb A_2_ (%)	Hb Q-Thailand (%)	Hb QA_2_ (%)
Hb Q-Thailand not associated with the -α^4.2^ deletion									
αα/αα^CD74 GAC>CAC^, β/β	7	26.3 ± 3.8	127.1 ± 18.1	86.7 ± 5.2	28.4 ± 1.6	80.1 ± 0.5	2.6 ± 0.3	16.6 ± 0.4	0.6 ± 0.1
-α^3.7^/αα^CD74 GAC>CAC^, β/β	2	26.5 ± 6.4	128 ± 8.5	79.2 ± 10.0	28.5 ± 2.9	77.0 ± 3.7	2.7 ± 0	22.4 ± 0.4	0.9 ± 0.1
αα/αα^CD74 GAC>CAC^, β/β	1	1d	NA	NA	NA	20.1	0	65.7 (Hb Q-Thailand + Hb F)	13.2 (Hb QF)
Hb Q-Thailand associated with the -α^4.2^ deletion									
αα/-α^4.2&CD74 GAC>CAC^, β/β	8	25.9 ± 17.9	122.1 ± 18.0	75.6 ± 10.2	24.5 ± 3.6	68.0 ± 0.6	2.6 ± 0.3	28.2 ± 0.8	1.0 ± 0.1
-α^3.7^/-α^4.2&CD74 GAC>CAC^, β/β	1	28	149	70.2	23.2	55.2	1.7	42.0	1.2
αα/-α^4.2&CD74 GAC>CAC^ β^IVS−II−654M^/β^N^	1	1	101	58.5	19.2	63.2	4.1	27.5	1.07
αα/-α^4.2&CD74 GAC>CAC^, β/β	1	1d	NA	NA	NA	18.9	0	65.7 (Hb Q-Thailand + Hb F)	22.7 (Hb QF)
Others									
αα/αα^CD9 AAC>AAA^, β/β	1	37	156	84.6	28	73.2	2.2	Hb Zhaoqing 23.7	Hb Zhaoqing-A_2_ 0.9

NA: no test data available.

### Genotype and phenotype characteristics of Hb Q-Thailand not linked to the -α^4.2^ deletion

Among the 22 samples confirmed by genetic analysis, 10 samples were found to be non-linked to the -α^4.2^ deletion in Hb Q-Thailand. Unexpectedly, two cases of Hb Q-Thailand not linked to the -α^4.2^ deletion co-inherited the -α^3.7^ deletion (Table 2). Hematological parameters were within normal ranges in the vast majority of samples, with the exception of three individuals exhibiting reduced MCV and MCH values: two heterozygous for Hb Q-Thailand (No.6 and No.7) and one (No.9) with co-inherited -α^3.7^ deletion (Table 2). The Hb Q-Thailand level in heterozygotes (16.6% ± 0.4%) was lower than that in individuals co-inheriting the -α^3.7^ deletion (22.4% ± 0.4%). Except for case No. 3 (Hb A_2_ 2.2%), Hb A_2_ levels fell within the normal range in all remaining cases. Additionally, a faint peak corresponding to Hb Q-Thailand-A_2_ (Hb QA_2_) was detected in Zone 3 (quantified as the percentage of the band relative to total Hb). Given that cord blood predominantly consists of Hb F, the presence of Hb Q-Thailand could not be directly ascertained in neonatal samples. Instead, an abnormal peak with elevated intensity was observed in Zone 5, which was identified as Hb Q-Thailand-F (Hb QF).

**TABLE 2:** The phenotypes and genotypes of patients with Hb Q-Thailand not linked to the -α^4.2^ deletion.

No	Gender	Age (years)	Hb (g/dL)	MCV (fL)	MCH (pg)	HbA (%)	Hb A_2_ (%)	Hb Q-Thailand (%)	Hb QA_2_ (%)	Genotypes
1	Female	22	128	83.1	28.4	80.0	2.9	16.4	0.7	αα/αα^CD74 GAC>CAC^, β/β
2	Female	21	94	84.4	28.7	79.6	3.0	16.8	0.6	αα/αα^CD74 GAC>CAC^, β/β
3	Male	29	154	97.6	31.2	80.8	2.2	16.3	0.8	αα/αα^CD74 GAC>CAC^, β/β
4	Female	27	118	86.2	28.6	79.4	2.4	17.3	0.6	αα/αα^CD74 GAC>CAC^, β/β
5	Female	26	133	86.8	28.9	80.2	2.5	16.8	0.5	αα/αα^CD74 GAC>CAC^, β/β
6	Female	27	131	86.8	26.5	80.7	2.7	16.1	0.6	αα/αα^CD74 GAC>CAC^, β/β
7	Female	32	132	81.7	26.3	80.2	2.6	16.4	0.6	αα/αα^CD74 GAC>CAC^, β/β
8	Male	31	134	86.3	27.1	74.4	2.7	22.1	0.8	-α^3.7^/αα^CD74 GAC>CAC^, β/β
9	Female	22	122	72.1	23	79.7	2.6	22.7	0.9	-α^3.7^/αα^CD74 GAC>CAC^, β/β
10	Male	1d	NA	NA	NA	20.3	0	65.7 (Hb Q-Thailand + Hb F)	13.2 (Hb QF)	αα/αα^CD74 GAC>CAC^, β/β

NA: no test data available.

### Genotype and phenotype features of Hb Q-Thailand linked to the -α^4.2^ deletion

A total of 11 samples with Hb Q-Thailand linked to the -α^4.2^ deletion were detected. Among these, one case was found to co-inherit the -α^3.7^ deletion, and another was compound heterozygous with β-thalassemia IVS-II-654 C>T (Table 3). Within this group, only two individuals exhibited normal hematological phenotypes (No. 1 and No.2), while the remaining samples showed reduced MCV and MCH values. CE revealed that both Hb Q-Thailand (28.2% ± 0.8%) and Hb QA_2_ (1.0% ± 0.1%) levels were higher than those observed in the group where Hb Q-Thailand was not linked to the -α^4.2^ deletion. The highest Hb Q-Thailand and Hb QA_2_ levels were observed in the case co-inheriting both the -α^4.2^ and -α^3.7^ deletions. The individual with co-inherited β-thalassemia presented with a phenotype and electrophoretic profile analogous to those of mild β-thalassemia, characterized by elevated Hb A_2_ and reduced MCV/MCH.

**TABLE 3:** The phenotypes and genotypes of patients with Hb Q-Thailand linked to the -α^4.2^ deletion.

No	Gender	Age (years)	Hb (g/dL)	MCV (fL)	MCH (pg)	HbA (%)	Hb A_2_ (%)	Hb Q-Thailand (%)	Hb QA_2_ (%)	Genotypes
1	Male	32	136	92	31.2	68.7	2.5	27.9	1.0	αα/-α^4.2&CD74 GAC>CAC^, β/β
2	Male	58	147	87	28.1	68.5	2.1	28.4	1.0	αα/-α^4.2&CD74 GAC>CAC^, β/β
3	Female	3	112	71.3	23.8	67.2	2.8	29.0	1.0	αα/-α^4.2&CD74 GAC>CAC^, β/β
4	Male	9M	93	75.4	23.3	68.2	2.9	26.5	1.0	αα/-α^4.2&CD74 GAC>CAC^, β/β
5	Male	8	120	72.7	23.6	68.0	2.7	28.2	1.2	αα/-α^4.2&CD74 GAC>CAC^, β/β
6	Female	30	135	73.5	23.6	67.2	2.7	29.0	1.1	αα/-α^4.2&CD74 GAC>CAC^, β/β
7	Female	25	105	58.5	19.2	68.6	2.3	28.1	0.9	αα/-α^4.2&CD74 GAC>CAC^, β/β
8	Female	25	129	74.4	23.5	67.2	2.7	28.9	1.1	αα/-α^4.2&CD74 GAC>CAC^, β/β
9	Male	28	149	70.2	55.2	55.2	1.7	42.0	1.2	-α^3.7^/-α^4.2&CD74 GAC>CAC^, β/β
10	Male	1d	NA	NA	NA	18.9	0	65.7 (Hb Q-Thailand + Hb F)	22.7 (Hb QF)	αα/-α^4.2&CD74 GAC>CAC^, β/β
11	Female	1	101	58.5	19.2	63.2	4.1	27.5	1.1	αα/-α^4.2&CD74 GAC>CAC^, β^IVS−II−654M^/β^N^

NA: no test data available.

### Sanger sequencing of α-globin chain

**FIGURE 3:**
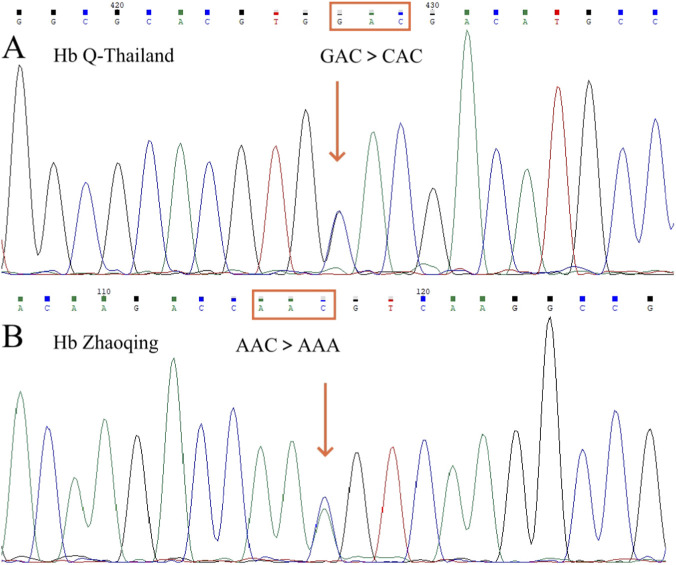
Sanger sequencing results: **(A)** Hb Q-Thailand, showing a GAC→CAC mutation at codon 74 of the *HBA*1 gene; **(B)** Hb Zhaoqing, showing an AAC→AAA mutation at codon 9 of the *HBA2* gene.

Of the 37 suspected positive samples identified through screening, 22 underwent conventional thalassemia genetic analysis and α-globin chain sequencing, with 21 confirmed to carry Hb Q-Thailand (*HBA1*:c.223G>C) (Figure 3A). Additionally, one rare variant, Hb Zhaoqing (*HBA2*:c.30C>A), was also detected (Figure 3B). This variant presented with an Hb Zhaoqing level of 23.7%, a slightly reduced Hb A_2_ value (2.2%), and a normal hematological phenotype.

## Discussion

To the best of our knowledge, Hb Q-Thailand has been considered to be almost invariably linked in cis with the -α^4.2^ deletion. However, findings from the Cenxi population in Guangxi have prompted a reassessment of this notion. Our screening results revealed a carrier rate of approximately 0.16% (37/23,546) for Hb Q-Thailand in this region. Among the 22 samples tested for genetic confirmation, 11 showed the expected linkage between Hb Q-Thailand and the -α^4.2^ deletion, whereas 10 showed Hb Q-Thailand without this deletion, accounting for 47.6% (10/21) of the confirmed cases. Additionally, one sample was identified as carrying Hb Zhaoqing, a rare variant not previously documented in the literature. This variant results from a nucleotide substitution (AAC>AAA) at codon 9 of the *HBA2* gene, leading to an amino acid change from asparagine to lysine.

The linkage between Hb Q-Thailand and the -α^4.2^ deletion represents a classic example of linkage disequilibrium in population genetics, although this association is not absolute. Several hypotheses may explain the mechanism underlying this observed linkage (Zeng et al., 1992; Singsanan et al., 2010; Panyasai and Pornprasert, 2014). First, the physical proximity of the two mutations on chromosome 16 likely plays a contributory role. The α2-and α1-globin genes are arranged in tandem within close proximity; the -α^4.2^ deletion, arising from unequal crossing-over, removes the entire α2-globin gene along with the 5′region of the α1-globin gene. Second, linkage disequilibrium may account for the persistence of this haplotype. Given the close physical proximity of these two genetic alterations within the same gene cluster and their relatively recent evolutionary origin, recombination events may not have had sufficient time to disrupt this combination. Third, an anthropological origin hypothesis suggests that the Hb Q-Thailand mutation may have initially arisen on a chromosome already carrying the -α^4.2^ deletion. Alternatively, at the time the -α^4.2^ deletion occurred, the remaining α1-globin gene may have coincidentally carried this mutation, thereby propagating this linked configuration throughout subsequent populations.

The observation that 47.6% of individuals in the Cenxi population carry Hb Q-Thailand genotypes unlinked to the -α^4.2^ deletion is particularly striking. This finding further substantiates that the linkage between these two genetic alterations is not absolute. The absence of linkage between Hb Q-Thailand and the -α^4.2^ deletion may be attributed to the high degree of homology between the α2-and α1-globin genes. During meiotic division, homologous chromosomes undergo pairing and crossing-over; although the -α^4.2^ deletion itself arises from unequal crossing-over events, subsequent unequal crossing-over or gene conversion events in carriers could potentially “copy” the Hb Q-Thailand from a deleted chromosome onto a normal chromosome, or alternatively, retain the mutation on a normal chromosome while exchanging away the deletion. The high proportion of such unlinked cases suggests that carriers in the Cenxi area may share a common ancestral origin. This phenomenon of rare variants exhibiting regional clustering has been previously documented; we have reported analogous findings regarding α-thalassemia fusion genes frequently detected in the Huadu District of Guangzhou (Huang et al., 2024).

Among the 10 cases in which Hb Q-Thailand was not linked to the -α^4.2^ deletion, 8 were identified as Hb Q-Thailand heterozygotes, while the remaining 2 exhibited co-inheritance of α^+^-thalassemia (-α^3.7^ deletion). No homozygous Hb Q-Thailand cases were identified in this study, nor was Hb Q-Thailand observed to be co-inherited with α^0^-thalassemia observed. In this group, the vast majority of samples demonstrated normal hematological phenotypes, with only three individuals exhibiting abnormal hematological parameters, suggesting that routine hematological analysis alone is insufficient for reliable identification of Hb Q-Thailand carriers. CE revealed that although only one sample showed reduced Hb A_2_ levels, all samples displayed elevated abnormal peaks in Zone 9, corresponding to the migration position of Hb Q. The Hb Q-Thailand levels were higher in individuals co-inheriting α^+^-thalassemia compared to those with simple heterozygosity. In peripheral blood samples, the presence of a variant peak exceeding 15% in Zone 9 together with a detectable Hb QA_2_ variant peak was strongly suggestive of Hb Q-Thailand. Notably, elevated abnormal peaks were also observed in cord blood samples from neonates with Hb Q-Thailand, demonstrating the utility of CE for neonatal screening. These findings indicate that CE provides a simple and reliable approach for Hb Q-Thailand screening. It is worth noting, however, that a rare variant, Hb Zhaoqing, was unexpectedly identified in the present study. This variant exhibits a normal hematological phenotype and migrates to the Hb F/Q region on CE. Nevertheless, its level is lower than that of Hb Q-Thailand, accompanied by a mildly decreased Hb A_2_ level. Collectively, these features allow its differentiation from Hb Q-Thailand.

In the group with Hb Q-Thailand linked to the -α^4.2^ deletion, one case was found to co-inherit the -α^3.7^ deletion, and another exhibited co-inheritance of β-thalassemia. With the exception of two peripheral blood samples that displayed normal hematological phenotypes, all other samples demonstrated reduced MCV and MCH values, with some cases presenting with overt anemia. This hematological presentation is likely attributable to the co-inheritance of α^+^-thalassemia (-α^4.2^ deletion) linked in cis with Hb Q-Thailand. Hb analysis revealed that six cases maintained Hb A_2_ levels within the normal reference range, while both Hb Q-Thailand and Hb QA_2_ levels were significantly elevated compared to those observed in the group where Hb Q-Thailand was not linked to the -α^4.2^ deletion. In the case with co-inherited β-thalassemia, the hematological phenotype closely resembled that of β-thalassemia trait, characterized by elevated Hb A_2_ and reduced MCV/MCH. When the α^+^-thalassemia was co-inherited, the Hb Q-Thailand level exceeded 40%, consistent with our previously reported cases (Liang et al., 2023). Our earlier investigations have further characterized the -α^4.2^ deletion linked to Hb Q-Thailand as the -α^4.2II^ subtype, whereas the more commonly encountered deletion in the general population is the -α^4.2I^ subtype (Liang et al., 2023).

In the present study, no co-inheritance with α^0^-thalassemia was detected in either the group with Hb Q-Thailand linked to the -α^4.2^ deletion or the group without this linkage. Previous studies have reported that Hb Q-Thailand linked to the -α^4.2^ deletion can co-inherit not only with other hemoglobin variants such as Hb E, Hb Tak, Hb J-Bangkok, Hb Leiden, and Hb CS, but also with various α- or β-thalassemia mutations (Panyasai et al., 2018; Jindadamrongwech et al., 2010; Jiang et al., 2016; Ren et al., 2025; He et al., 2017; Sanchaisuriya et al., 2005). Among these, co-inheritance with α^0^-thalassemia results in the most severe phenotype, diagnosed as Hb Q-H disease, which may present with moderate anemia. This severe manifestation is attributable to the linked -α^4.2^ deletion, which itself constitutes an α^+^-thalassemia defect. In contrast, when Hb Q-Thailand without the linked -α^4.2^ deletion co-occurs with α^0^-thalassemia, the condition should not be classified as Hb Q-H disease but rather as mild α-thalassemia. This distinction carries important clinical implications for genetic counseling. When one partner carries Hb Q-Thailand linked to the -α^4.2^ deletion and the other carries α^0^-thalassemia, informed consent and prenatal diagnosis are warranted due to the risk of Hb Q-H disease. Conversely, if one partner carries Hb Q-Thailand without the -α^4.2^ deletion and the other carries α^0^-thalassemia, prenatal diagnosis is not indicated, as the resulting phenotype would be mild.

Several limitations should be acknowledged in this study. First, based on the consensus in the literature, we directly assumed that Hb Q-Thailand is inherited in cis with the -α^4.2^ deletion; therefore, we did not perform cis-trans configuration analysis on all samples. Second, despite conducting a large-cohort screening, the rarity of Hb Q-Thailand not linked in cis with the -α^4.2^ deletion led to a low number of detected cases. Third, the limited sample size inherently reduced the likelihood of detecting co-inherited thalassemia mutations, making it impossible to include a broader range of compound heterozygous samples. Finally, due to the scarcity of such cases and the consequent lack of compound heterozygotes, the relationship between genotype and phenotype could not be fully characterized, thereby limiting the provision of more robust and reliable clinical references. To comprehend the precise relationship between genotype and phenotype, it is necessary to collect additional samples for further study.

## Data Availability

The original contributions presented in the study are included in the article/supplementary material, further inquiries can be directed to the corresponding author.
